# Relationship between sodium level and in-hospital mortality in traumatic brain injury patients of MIMIC IV database

**DOI:** 10.3389/fneur.2024.1349710

**Published:** 2024-03-18

**Authors:** Xiaoliang Wang, Xin Li, Jiahao Sun, Mengmeng Wang, Wenjuan Lang, Xin Xu

**Affiliations:** ^1^Neurology Department of Qingdao Municipal Hospital, Nanjing Medical University, Qingdao, Shandong Province, China; ^2^Neurology Department of Affiliated Hospital of Qingdao University, Qingdao, Shandong Province, China

**Keywords:** traumatic brain injury, hypernatremia, mortality, MIMIC IV database, ICU

## Abstract

**Background:**

An association between prognosis and high sodium levels in Traumatic Brain Injury (TBI) patients in Intensive Care Units (ICUs) has been noted, but limited research exists on the ideal sodium level in these patients or the impact on early mortality, using the MIMIC-IV database.

**Methods:**

A retrospective survey was conducted on TBI patients from the MIMIC-IV database. Patients were divided into two categories based on their highest serum sodium level within 24 h of admission exceeding 145 mmol/L: those with hypernatremia, and those with moderate-to-low sodium levels. Collected covariates encompasses demographic, clinical, laboratory, and intervention variables. A multivariate logistic regression model was implemented to forecast in-hospital mortality.

**Results:**

The study included 1749 TBI patients, with 209 (11.5%) experiencing in-hospital deaths. A non-linear test exposed an L-shaped correlation between sodium level and in-hospital mortality, with mortality rates increasing after a turning point at 144.1 mmol/L. Compared to the moderate-to-low group’s 9.3% mortality rate, the hypernatremia group had a significantly higher mortality rate of 25.3% (crude odds ratio = 3.32, 95% confidence interval: 2.37 ~ 4.64, *p* < 0.001). After adjusting for all covariates, the hypernatremia group continued to show a significant correlation with higher mortality risk (adjusted odds ratio = 2.19, 95% confidence interval: 1.38 ~ 3.47, *p* = 0.001). This trend remained consistent regardless of the analyses stratification.

**Conclusion:**

The study reveals an L-shaped relationship between sodium levels and in-hospital deaths, with a pivotal point at 144.1 mmol/L. TBI patients displaying hypernatremia were independently linked to higher in-hospital mortality, underlining the need for further studies into targeted management of sodium levels in these patients.

## Introduction

Traumatic brain injury (TBI) affect nearly 69 million people per year, often resulting in long-lasting disability and mortality (1). Despite the pervasiveness of this issue, effective treatments for TBI are limited. Hypernatremia is commonly identified in TBI patients admitted to Intensive Care Units (ICUs) (2). The mechanisms contributing to hypernatremia in these patients are complex, encompassing central diabetes insipidus, osmotic agent administration, high sodium infusion and imbalance of fluid intake and output (3). Several studies suggest an independent correlation between hypernatremia and mortality following severe TBI (3, 4). The causal relationship, however, remains indistinct as hypernatremia can indicate both disease severity and a therapeutic target for fundamental medication such as mannitol and/or hypertonic saline (5). Additional research is imperative to understand the prognostic implication of sodium levels in TBI patients and to clarify the optimal sodium level for these patients during ICU admission although the causal relationship cannot be established. The aim of the current study is to evaluate the impact of sodium levels on in-hospital mortality among patients with severe TBI.

## Method

### Study population

This retrospective study exploits patient data from the Medical Information Mart for Intensive Care-IV (MIMIC-IV) cohort, a single-center, longitudinal cohort from 2008 to 2019. The MIMIC-IV database comprises diverse patient information from ICUs. We completed a training programme facilitated by the PhysioNet team and secured official approval to use the MIMIC-IV database from the review boards of the “Massachusetts Institute of Technology and Beth Israel Deaconess Medical Center” (ID: 11744558). Because the patient data utilized in this study was anonymized within the database, informed consent was unnecessary. The data extraction code, available on GitHub^1^ (6), was employed, and the study was conducted in adherence to the STROBE (“Strengthening the Reporting of Observational Studies in Epidemiology”) guidelines (7).

### Inclusion and exclusion criteria

TBI patient data extracted from the database using the diagnostic codes of ICD-9 or ICD-10 (International Classification of Disease, Ninth and Tenth Versions; ICD-9: 85*; ICD-10: S06*) were included in this study. The criteria defined for inclusion were: (1) patients aged 18 years or older; (2) patients with an ICU length of stay (LOS) lasting 24 h or more; and (3) consideration only of the initial ICU stay record. Meanwhile, the exclusion criteria consisted of: (1) patients younger than 18 years and (2) patients with an ICU stay less than 24 h.

### Data extraction

Structured Query Language (SQL) was employed for data extraction. Variables, collected within 24 h of ICU admission, were extracted. If repeated test results presented themselves, only the worst was selected. These variables include:

1. Basic patient characteristics such as sex, admission age, race, admission time, ICD code, and in-hospital death.
2. Vital signs like temperature, mean blood pressure (MBP), heart rate, and SpO2.
3. Illness severity scores, which included the SOFA score, SAPS II, GCS, and Charlson comorbidity index.
4. Laboratory results such as WBC count, creatinine levels, HCT, etc.
5. Treatment methods, such as mechanical ventilation, hypersaline infusion.
6. Comorbidities—myocardial infarction, peripheral vascular disease, dementia, COPD, malignant cancer, renal disease, and severe liver disease, among others.

### Variable definition and outcomes

The Hypernatremia group was characterized by a sodium level exceeding 145 mmoL/L during the first 24 h after ICU admission, while the moderate to low group had a sodium level of 145 mmoL/L or below. The primary objective of this study was to explore in-hospital mortality as the chief outcome.

### Statistical analysis

Patient baseline characteristics were stratified by differing sodium level groups. Continuous data were represented as either mean ± standard deviation or median (inter-quartile range), while categorical variables were depicted as numbers (percentages) where suitable. Statistical comparisons between the two groups employed the chi-square test or Fisher’s exact test for categorical variables, and continuous variables were assessed via the analysis of variance test or rank-sum test. No imputation methods were used for missing data. Patients with missing data (with a missing rate below 1%) on variables like mean glucose level, WBC, BUN, and GCS were excluded. If a patient was intubated and could not provide a verbal score for the Glasgow Coma Scale (GCS), it was estimated from the eye and motor scores, as reported in previous studies (8).

Univariable logistic regression was utilized to assess the potential effect of sodium levels on in-hospital-death and to screen for confounders. To further analyze the relationship between Hypernatremia and in-hospital death, a multivariable logistic regression model was adopted, with models adjusted by potential confounders.

A restricted cubic spline was applied to investigate potential nonlinear relationships between sodium level and in-hospital mortality, adjusting the analysis for factors including age, gender, MBP, heart rate, spo2, glucose, platelets, hematocrit, hemoglobin, WBC, potassium, BUN, creatinine, Charlson comorbidity index, SAPSII, GCS, and length of stay (LOS) in ICU. Inflection point analysis was undertaken to identify the optimal serum sodium level corresponding to the lowest mortality. A likelihood ratio test was conducted to gauge how closely in-hospital death and sodium levels were related. Heterogeneity across subgroups was examined through logistic proportional hazards analysis, and a likelihood ratio test evaluated the interactions.

A two-tailed test with a threshold of *p* < 0.05 determined statistical significance. The data were analyzed using the R software package (R version 4.0.3) and Free Statistics software version 1.7.

## Result

### Baseline characteristics

**Figure 1:**
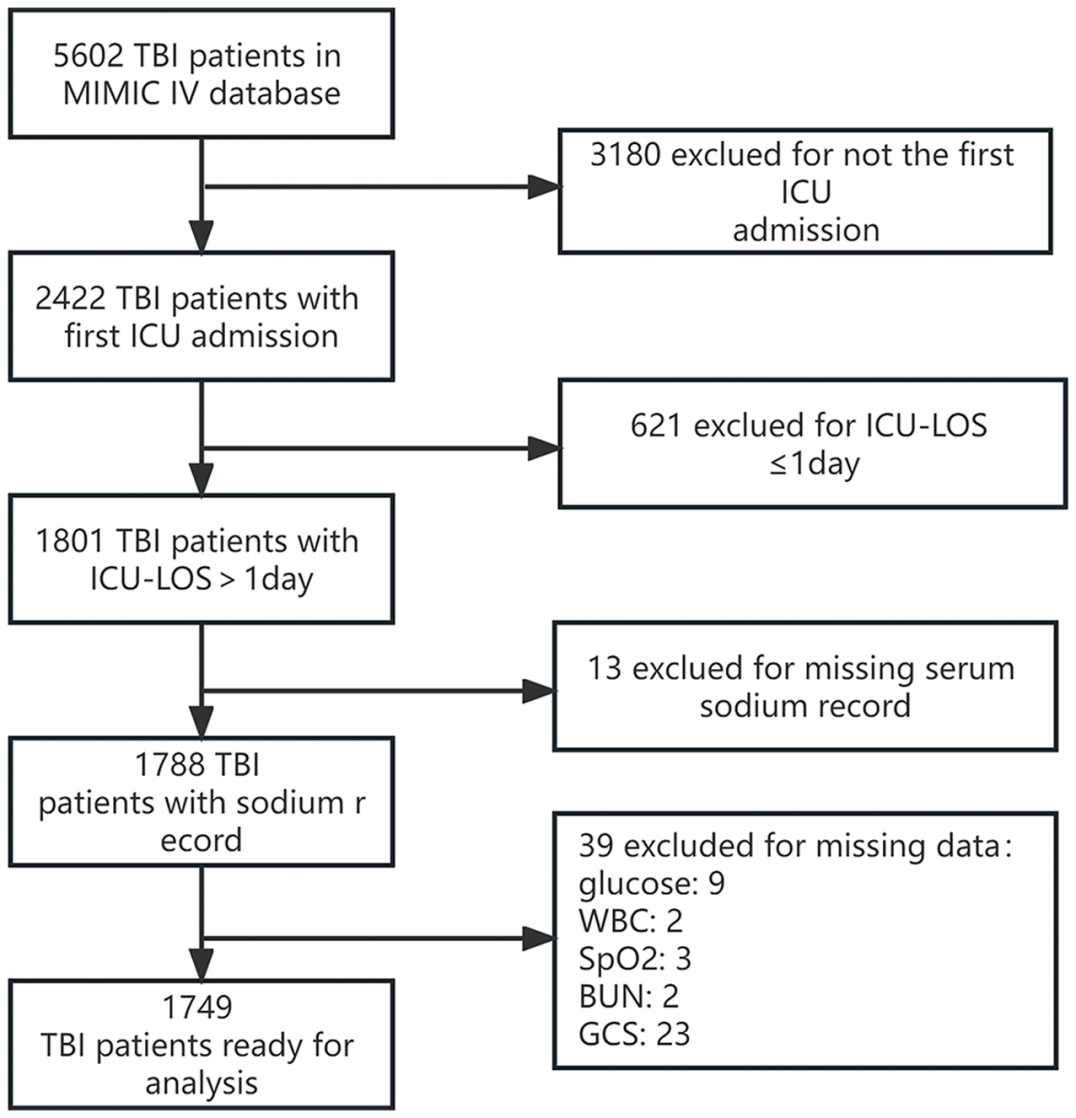
Detailed flowchart illustrating the participant recruitment process. GCS, Glasgow Coma Scale; WBC, white blood cell count; ICU, intensive care unit.

We identified 2,422 individuals diagnosed with Traumatic Brain Injury (TBI) who were first admitted to the ICU. Out of these, 421 patients had an ICU stay shorter than one day. With missing data cases excluded, 1,749 records remained for our final cohort analysis. The process of participant admission and exclusion is depicted in Figure 1.

Table 1 outlines the clinical characteristics of the selected participants. The cohort comprised of 1,749 patients, with an average age of 65.9 ± 20.3 years. The majority were males (62.1%; *n* = 1,087). A total of 202 patients (11.5%) did not survive their hospital stay. We classified 249 patients with higher sodium levels into a hypernatremia group. This group showed younger patient age and lesser prevalence of comorbidities such as malignant cancer but higher White Blood Cell (WBC) count and potassium levels. The Simplified Acute Physiology Score II (SAPSII) was higher and Glasgow Coma Scale (GCS) lower in the hypernatremia group, which also contained most of the mechanically ventilated patients. More frequently administered with hypertonic saline, the hypernatremia group had a longer ICU-Length Of Stay (LOS) and a higher mortality rate compared to the moderate-to-low group (25.3% vs. 9.3%). Refer to Table 1 for details.

**Table 1:** Characteristic of patients categorized by sodium level.

	Total (*n* = 1749)	Moderate to low group (*n* = 1,500)	Hypernatremia group (*n* = 249)	*p*
Demographics				
Age, Mean (SD), year	65.9 (20.3)	66.8 (20.0)	60.7 (21.3)	**<0.001**
Sex, *n* (%)				0.647
Male	1,087 (62.1)	929 (61.9)	158 (63.5)	
Female	662 (37.9)	571 (38.1)	91 (36.5)	
Vital signs, Mean (SD)				
MBP, mmHg	81.9 (10.1)	81.8 (10.1)	82.6 (10.1)	0.227
temperature, °C	37.0 (0.5)	37.0 (0.5)	37.1 (0.7)	**0.006**
SpO2, %	97.4 (1.7)	97.4 (1.7)	97.8 (1.8)	**<0.001**
Lab test, Mean (SD)				
Glucose, mg/dl	132.8 (40.8)	132.5 (40.6)	134.8 (42.2)	0.396
Hematocrit, %	36.8 (5.7)	36.8 (5.6)	37.3 (5.9)	0.2
Hemoglobin, g/dL	12.3 (2.0)	12.3 (2.0)	12.3 (2.1)	0.948
Platelets, 10^9^/L	221.8 (97.3)	223.4 (98.3)	212.1 (91.1)	0.092
WBC, 10^9^/L	12.9 (7.4)	12.7 (7.5)	14.1 (7.0)	**0.004**
BUN, mg/dl	20.8 (14.1)	20.7 (14.2)	21.4 (13.3)	0.451
Creatinine, mg/dl	1.2 (1.0)	1.2 (1.0)	1.2 (0.8)	0.331
Potassium, mmol/L	4.4 (0.8)	4.4 (0.8)	4.5 (0.8)	0.023
Sodium, mmol/L	140.6 (4.8)	139.4 (3.5)	147.7 (4.9)	**<0.001**
Severity score, Mean (SD)				
charlson_comorbidity_index	4.3 (2.8)	4.4 (2.8)	3.9 (2.9)	**0.004**
SAPSII	33.4 (11.8)	32.8 (11.6)	36.9 (12.5)	**<0.001**
GCS	11.1 (3.6)	11.4 (3.4)	9.4 (4.2)	**<0.001**
Commodities, *n* (%)				
myocardial_infarct	140 (8.0)	122 (8.1)	18 (7.2)	0.626
congestive_heart_failure	232 (13.3)	198 (13.2)	34 (13.7)	0.845
Diabetes	300 (17.2)	267 (17.8)	33 (13.3)	0.078
renal_disease	214 (12.2)	185 (12.3)	29 (11.6)	0.759
malignant_cancer	74 (4.2)	70 (4.7)	4 (1.6)	**0.026**
Interventions, *n* (%)				
Ventilation	711 (40.7)	556 (37.1)	155 (62.2)	**<0.001**
Hypertonic saline	112 (6.4)	74 (4.9)	38 (15.3)	**<0.001**
Brain surgery	398 (22.8)	343 (22.9)	55 (22.1)	0.786
Outcomes				
In-hospital death, *n* (%)	202 (11.5)	139 (9.3)	63 (25.3)	**<0.001**
LOS-ICU, Mean(SD)	4.9 (6.0)	4.5 (5.2)	7.1 (9.3)	**<0.001**

SAPS, simplified acute physiology score; COPD, chronic obstructive pulmonary disease; MBP, mean blood pressure; WBC, white blood cell count; BUN, blood urea nitrogen. GCS, Glasgow coma scale; LOS-ICU, length of stay in intensive care unit; SD, standard derivation; SASPII, Simplified Acute Physiology Score II. Bold values indicate statistical significance (*p* < 0.05).

### Relationship between sodium level on in-hospital mortality

Our univariate logistic regression analysis (Table 2) indicates an increasing in-hospital mortality in line with rising sodium levels. Specifically, each unit (mmol/L) increase in sodium level corresponded to a 10% rise in the odds of in-hospital death (OR:1.1; 95%CI:1.071.14; *p* < 0.001). The hypernatremia group exhibited a 3.32 times higher mortality rate relative to the reference group (crude odds ratio = 3.32, 95% confidence interval: 2.374.64, *p* < 0.001).

**Table 2:** Univariate logistic regression evaluating the association between baseline characteristics and in-hospital mortality.

Variable	OR_95CI	*p*_value
Sodium level	1.1 (1.07 ~ 1.14)	**<0.001**
Hypernatremia group	3.32 (2.37 ~ 4.64)	**<0.001**
Sex	1.19 (0.89 ~ 1.61)	0.245
Age	1.02 (1.01 ~ 1.03)	**<0.001**
GCS	0.67 (0.63 ~ 0.7)	**<0.001**
Heart_rate	1.02 (1.01 ~ 1.03)	**<0.001**
MBP	0.97 (0.96 ~ 0.99)	**<0.001**
SpO2	1.31 (1.19 ~ 1.45)	**<0.001**
Glucose	1.01 (1 ~ 1.01)	**<0.001**
Hematocrit	0.95 (0.92 ~ 0.97)	**<0.001**
Hemoglobin	0.85 (0.79 ~ 0.92)	**<0.001**
Platelets	1 (1 ~ 1)	0.236
WBC	1.03 (1.01 ~ 1.05)	**0.001**
BUN	1.03 (1.02 ~ 1.03)	**<0.001**
Bicarbonate	0.94 (0.9 ~ 0.98)	**0.006**
Creatinine	1.19 (1.06 ~ 1.32)	**0.002**
Charlson comorbidity index	1.14 (1.08 ~ 1.2)	**<0.001**
SAPSII	1.08 (1.07 ~ 1.1)	**<0.001**
Ventilation	6.06 (4.29 ~ 8.56)	**<0.001**
Brain surgery	1 (0.71 ~ 1.42)	0.995
Hypertonic saline	2.96 (1.88 ~ 4.64)	**<0.001**

SAPS, simplified acute physiology score; COPD, chronic obstructive pulmonary disease; MBP, mean blood pressure; WBC, white blood cell count; BUN, blood urea nitrogen. GCS, Glasgow coma scale; SASPII, Simplified Acute Physiology Score II. Bold values indicate statistical significance (*p* < 0.05).

After accounting for confounding factors in the multivariate logistic models (Table 3), patients in the hypernatremia group were found to have 2.17 times the risk of in-hospital mortality compared to the reference group (adjusted odds ratio = 2.17, 95% confidence interval: 1.36 ~ 3.46, *p* = 0.001).

**Table 3:** Multivariable logistic regression models evaluating the association between hypernatremia and in-hospital mortality.

Model	crude.OR_95CI	crude.*p*_value	adj.OR_95CI	adj.*p*_value
Model 1	3.32 (2.37 ~ 4.64)	<0.001	3.85 (2.72 ~ 5.44)	<0.001
Model 2			2.33 (1.5 ~ 3.6)	<0.001
Model 3			2.28 (1.44 ~ 3.6)	<0.001
Model 4			2.17 (1.36 ~ 3.46)	0.001

Model 1: Adjusted for sex and age.

Model 2: Adjusted for Model 1 + GCS, Charlson comorbidity index and SASPII.

Model 3: Adjusted for Model2 + heart rate + MBP+ SpO2+ hematocrit + hemoglobin + platelets + WBC+ BUN+ bicarbonate + Creatinine + glucose.

Model 4:Adjuste for Model3+ ventilation, hypertonic saline and brain surgery.

OR, odd ratio; CI, confident interval.

**Figure 2:**
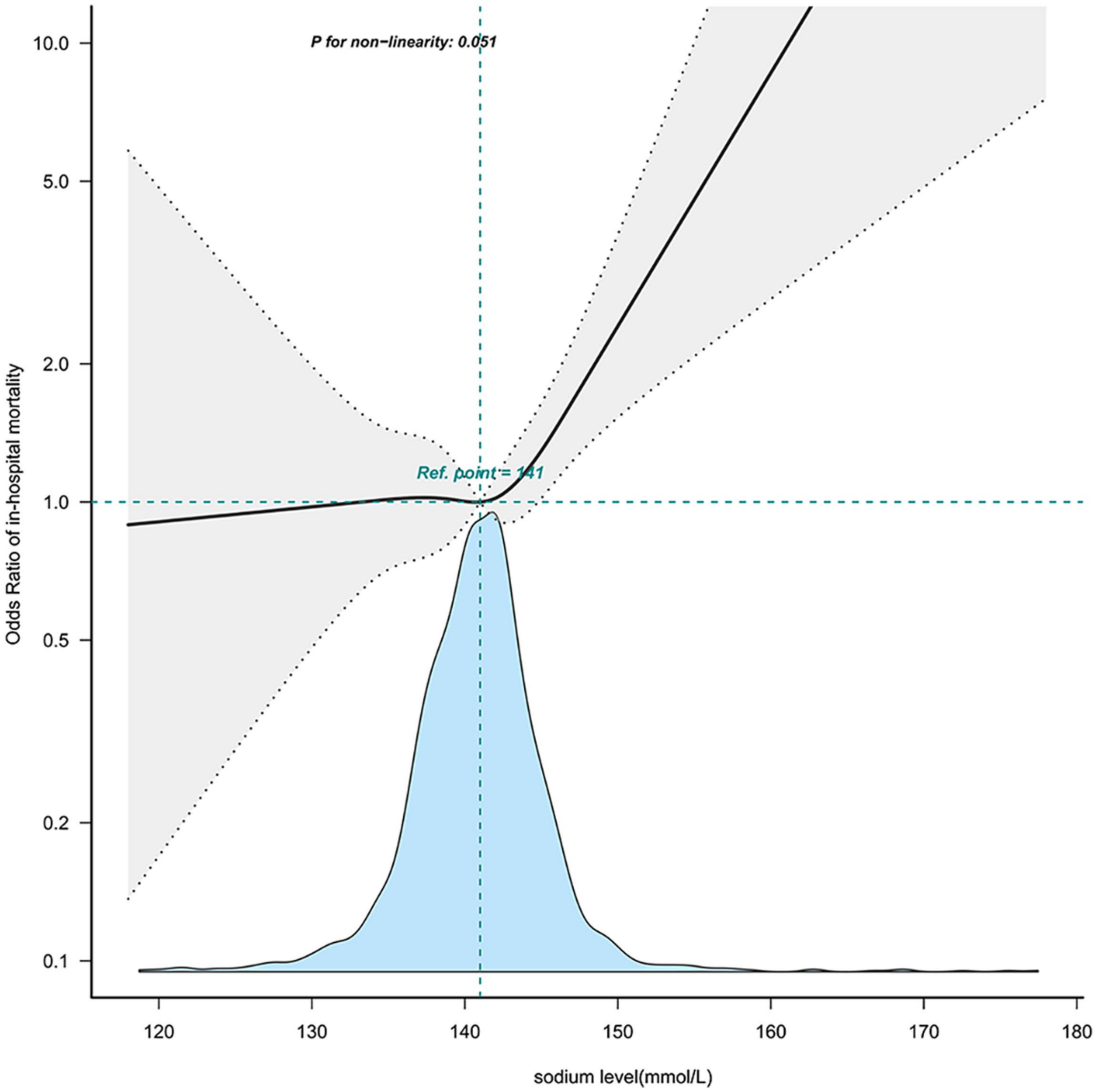
The nonlinear relationship between sodium level and in-hospital mortality. This curve illustrates the mortality fluctuation across varying sodium levels within the cohort, using the median level of 141 mmol/L as a reference point. The curve notably demonstrates a significant uptick in mortality commencing within the 141–150 mmol/L sodium range (P for non − linearity = 0.051). Adjusted sex, age, stroke type, GCS, heart_rate, MBP, SpO2, hematocrit, hemoglobin, platelet, WBC, BUN, bicarbonate, creatinine, Charlson_comorbidity_index, SAPSii, glucose, ventilation status. GCS, Glasgow Coma Scale; WBC, white blood cell count; BUN, blood urea nitrogen; MBP, mean blood pressure.

Further investigation into the relationship between sodium levels and in-hospital mortality was conducted through multivariable-adjusted restricted cubic spline analyses. There emerged a J-shaped association, indicative of a non-linear relationship between sodium levels and mortality (Figure 2). The inflection point for sodium levels was about 144.1 mmol/L (95% confidence interval: 143.505 ~ 144.785, *p* = 0.05) (Table 4). Beyond this threshold, every additional 1 mmol/L of sodium level led to a 9.7% rise in in-hospital mortality odds (OR = 1.097; 95%CI:1.002 ~ 1.202, *p* = 0.05). Below this point, sodium level increases did not impact in-hospital mortality rates (OR = 0.992 (0.936 ~ 1.051), *p* = 0.7856).

**Table 4:** The nonlinear relationship between sodium level and in-hospital mortality.

Threshold	OR(95% CI)	*p*
Threshold point	144.145 (143.505,144.785)	–
Slope<144.145	0.992 (0.936 ~ 1.051)	0.7856
Slope ≥ 144.145	1.097 (1.002 ~ 1.202)	0.0459
Likelihood ratio test	–	0.05

Adjusted: sex + age + GCS+ heart rate + MBP+ SpO2+ hematocrit + hemoglobin + platelets + WBC+ BUN+ bicarbonate + creatinine + Charlson comorbidity index + glucose + ventilation + SASPII+ brain surgery. OR, odd ratio; CI, confident interval.

### Sensitivity analysis

**Figure 3:**
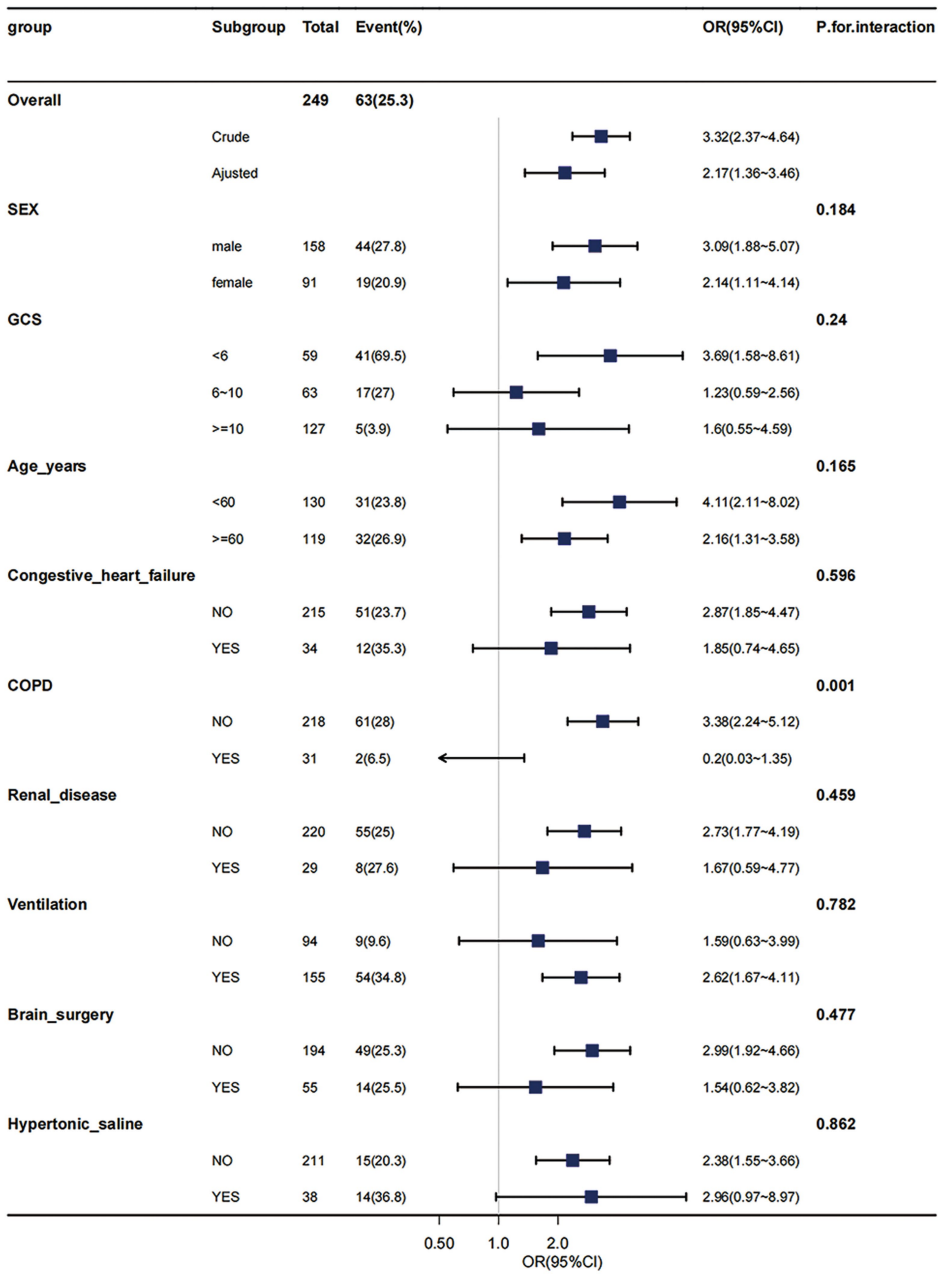
Effect size of hypernatremia on in-hospital mortality in different subgroups. Subgroup analyses were executed to assess patients with varied demographics, including age, gender, Glasgow Coma Scale (GCS) scores, and the presence of comorbid conditions such as Chronic Obstructive Pulmonary Disease (COPD), congestive heart failure, and renal disease. These analyses were conducted within the hypernatremia group (comprising 249 patients) and compared with those in the moderate-to-low sodium level group. The results from this subgroup analyses have demonstrated consistent and robust findings. COPD, chronic obstructive pulmonary disease; GCS, Glasgow Coma Scale, WBC, white blood cell count; BUN, blood urea nitrogen. Adjusted: MBP, heart rate, SpO2, hematocrit, hemoglobin, platelets, WBC, BUN, bicarbonate, creatinine, Charlson comorbidity index, SAPSii, glucose.

Through multivariable logistic models, we carefully adjusted for diverse confounding factors to gauge the relationship between sodium levels and in-hospital mortality. Intriguingly, this association remained consistent across all models (refer to Table 3). To validate the resilience of our results, subgroup analyses were performed based on a variety of confounding variables such as age, gender, GCS, and comorbidities like COPD, congestive heart failure, and renal disease (depicted in Figure 3). These analyses reaffirmed our preliminary findings. The potential interaction within the COPD subgroup could be attributed to its relatively small size. No significant interactions were detected in any other sub-groups (all *p* > 0.05) (see Figure 3).

To delve deeper into the relationship between sodium levels and in-hospital mortality, we reclassified our cohort into three categories: the hyponatremia group (*n* = 117), which includes patients with sodium levels below 135 mmol/L; the hypernatremia group (*n* = 249), comprising patients with sodium levels above 145 mmol/L; and the normonatremia group (*n* = 1,383), consisting of patients with sodium levels ranging from 135 to 145 mmol/L. We employed both univariate and multivariate logistic regression analyses to examine this association, as presented in Table 5. Compared to the normonatremia group, the hyponatremia group did not exhibit a significant association with in-hospital mortality (crude OR = 1.02, 95% CI: 0.53–1.94, *p* = 0.958; adjusted OR = 0.75, 95% CI: 0.34–1.63, *p* = 0.466). In contrast, the hypernatremia group demonstrated a significant association with increased in-hospital mortality (crude OR = 3.32, 95% CI: 2.37–4.66, *p* < 0.001; adjusted OR = 2.11, 95% CI: 1.32–3.38, *p* = 0.002).

**Table 5:** The association between different sodium levels with in-hospital mortality.

Variable	n.total	n.event_%	crude.OR_95CI	crude.*p*_value	adj.OR_95CI	adj.*p*_value
Normal group	1,383	128 (9.3)	1 (Ref)		1 (Ref)	
Hyponatremia group	117	11 (9.4)	1.02 (0.53 ~ 1.94)	0.958	0.75 (0.34 ~ 1.63)	0.466
Hypernatremia group	249	63 (25.3)	3.32 (2.37 ~ 4.66)	<0.001	2.11 (1.32 ~ 3.38)	0.002

Mutivriable logistic regression used variables adjusted in model 4 showed in Table 3. OR, odd ratio; CI, confident interval.

## Discussion

In this investigation of adult Traumatic Brain Injury (TBI) patients from MIMIC-IV, we observed a higher in-hospital mortality rate for those patients with hypernatremia. Restricted Cubic Splines (RCS) analysis revealed an L-shaped dose–response correlation between serum sodium concentration and mortality risk. We determined the ideal serum sodium concentration for TBI patients to be ≤144.1 mmol/L. Our study reveals that hypernatremia independently correlates with mortality during hospitalization and that a serum sodium level of ≤144.1 mmol/L might be an effective target for TBI treatment management. To the best of our understanding, this report distinguishes itself as a pioneering effort in establishing a patient-specific threshold for serum sodium levels, exclusive to Traumatic Brain Injury (TBI) patients admitted to the Intensive Care Unit (ICU).

Hypernatremia, often induced by water loss due to ventilation, sedation therapy, or osmotic diuretic administration, frequently occurs in TBI patients (9). The use of hypertonic saline and mannitol to manage intracranial pressure commonly exacerbates hypernatremia especially when it becomes a treatment target for elevated ICP (5, 10). Additionally, hyperosmolar therapy-related acute kidney injury can intensify hypernatremia (3). It can also result from hypothalamic or pituitary gland dysfunction post-TBI, known as central diabetes insipidus, potentially indicating disease severity (5).

Maggiore et al. corroborated the association between the prevalence of hypernatremia and increased mortality rates in individuals suffering from severe traumatic brain injury. They identified that the emergence of hypernatremia was, in part, attributable to central diabetes insipidus (4).

We acknowledge that our study has the significant limitation like previous study’s that we also cannot establish causal relationship between hypernatremia and outcome. In an expansive healthcare database encompassing data from over 90,000 patients with traumatic brain injury (TBI), there was a clear correlation between hypernatremia and less favorable outcomes in this patient population (11). Previous studies reported hypernatremia and mortality associations in various neurological critical conditions. In neurologic ICUs, severe hypernatremia (serum sodium >160 mmol/L) was independently linked to in-hospital mortality (12). This observation was consistent with findings in pediatric study focusing on severe traumatic brain injury(TBI) (13). Serum sodium concentration has been used to predict ICU deaths in the neurological ICU, with an optimal cut-off at 147.55 mmol/L (14). Takahiro et al. found an L-shaped dose–response association between serum sodium concentration and mortality in cerebrovascular diseases, identifying a 147 mmol/L maximum threshold through restricted cubic splines analysis (15). These findings align with our study, though the slightly higher cut-off value may result from the different population studied. The discrepancy between the results observed by Tan and colleagues and our own can primarily be attributed to differences in patient demographics and injury severity within their study (16). Specifically, patients in their research cohort presented with a more severe average Glasgow Coma Scale (GCS) score (11.1 compared to our cohort’s 6) and were of a younger median age (52 versus 34 years, respectively). Recent meta-analyses further bolster the conclusion that hypernatremia is associated with heightened mortality risk (17).

The reason of hypernatremia on higher mortality can be listed as follows: Hypernatraemia is associated with various neuromuscular manifestations, such as muscle weakness, that can prolong the length of ICU stay and duration of mechanical ventilation which may increase mortality (18). Hypernatremia can lead to organ dysfunction, including abnormal hepatic function, disturbance of insulin resistance, cardiac dysfunction and muscle injury (14, 19). Hypernatremia can injure myelin and even cause secondary brain injury by increased cellular dehydration and decreased cerebral edema (3).

Mild hypernatremia induced by hyperosmolar agents that can effectively decrease ICP may result in a favorable prognosis (20, 21). Thus, maintaining sodium levels within an acceptable range is crucial. In our study, we determined the optimal target serum sodium concentration for TBI patients to be ≤144.1 mmol/L, offering a valuable therapeutic outlook for TBI. The lack of a lower sodium level threshold observation could be due to the inclusion of data within 24 h of ICU admission since hyponatremia often occurs days after TBI (22).

This study had several limitations. Primarily, the dataset’s nature prevented us from ruling out the specific etiology of hypernatremia, or adjusting properly for baseline factor influences on hypernatremia. This study is constrained by the limitation that in-hospital mortality was the sole outcome variable utilized in our analyses. More significant outcome measures, such as the Glasgow Outcome Scale-Extended (GOSE), which provide a more comprehensive evaluation of patient functionality beyond mortality, could not be obtained from the MIMIC-IV database. Another critical limitation of our research is the absence of intracranial pressure (ICP) values, which are a pivotal confounder for analysis. The omission of these values is due to an excessively high percentage of missing data, rendering their use infeasible for our study. Also, being a retrospective study, the test timing was not randomly planned potentially leading to the exclusion of some hypernatremic patients and the failure to use sodium over time to analyze the association to the outcome. Furthermore, MIMIC-IV being a single-center cohort may inherently carry biases related to specific medical practice styles. Therefore, validating our findings across different populations would require prospective studies in multiple ICUs.

## Conclusion

The current investigation conclusively establishes hypernatraemia in 24 h as a mortality marker in critically ill TBI patients. Additionally, it designates a specific cut-off point for serum sodium concentration at 144.1 mEq/L, beyond which the mortality risk escalates. These insights are instrumental for healthcare professionals tending to such critically ill TBI individuals.

## Data availability statement

The datasets presented in this article are not readily available because the data utilized in this study were obtained from the Medical Information Mart for Intensive Care IV (MIMIC-IV) Clinical Database (23). Requests for access to these datasets should be directed to PhysioNet (24, 25). Requests to access the datasets should be directed to www.physionet.org/.

## Ethics statement

The studies involving humans were approved by review boards of the “Massachusetts Institute of Technology and Beth Israel Deaconess Medical Center.” The studies were conducted in accordance with the local legislation and institutional requirements. The ethics committee/institutional review board waived the requirement of written informed consent for participation from the participants or the participants’ legal guardians/next of kin because Written consent from participants was waived, as the data used in this study adhered to anonymity requirements outlined by institutional protocols and national laws.

## Author contributions

XW: Conceptualization, Data curation, Formal analysis, Methodology, Software, Writing – original draft. XL: Methodology, Supervision, Validation, Writing – review & editing. JS: Data curation, Formal analysis, Validation, Writing – review & editing. MW: Investigation, Supervision, Writing – review & editing. WL: Methodology, Validation, Visualization, Writing – review & editing. XX: Writing – review & editing.

## Footnotes

^1^http://github.com/MIT-LCP/mimic-iv
